# Effect of Aptamin C on NK Cell Activity and Cytotoxicity: A Randomized Placebo-Controlled Trial and In Vitro Comparison with Vitamin C

**DOI:** 10.3390/antiox15070796

**Published:** 2026-06-25

**Authors:** Hyovin Ahn, June Lee, Jeong-Ho Park, Jae Sang Barn, Yejin Kim, Jae Seung Kang

**Affiliations:** 1 Laboratory of Vitamin C and Antioxidant Immunology, Department of Anatomy and Cell Biology, Seoul National University College of Medicine, Seoul 03080, Republic of Korea; jahb1220@snu.ac.kr; 2 Institute of Allergy and Clinical Immunology, Medical Research Center, Seoul National University, Seoul 08826, Republic of Korea; 3 Nexmos, Inc., Yongin-si 16827, Republic of Korea; ratury87@gmail.com (J.L.); joyjura80@gmail.com (J.-H.P.); 4 BABOBAGI Plastic Surgery, 517 Nonhyeon-ro, Gangnam-gu, Seoul 06129, Republic of Korea; barn8088@naver.com; 5 Department of Research and Development, N Therapeutics Co., Ltd., Seoul 08813, Republic of Korea; 6 Artificial Intelligence Institute, Seoul National University, Seoul 08826, Republic of Korea; 7 Department of Applied Bioengineering, Graduate School of Convergence Science and Technology, Seoul National University, Seoul 08826, Republic of Korea

**Keywords:** NK cell, Aptamin C, Vitamin C, Cytotoxicity, Aptamer

## Abstract

Natural killer (NK) cells are crucial components of innate immunity and rapidly eliminate abnormal cells through ligand–receptor signaling without prior sensitization. Vitamin C is known to enhance NK cell function; however, its susceptibility to oxidation may limit its efficacy in NK cell activation. This study evaluated the efficacy of Aptamin C, a stabilized conjugate of vitamin C and an aptamer, in enhancing NK cell activation. In the in vivo randomized placebo-controlled study, 120 participants were randomized to receive either Aptamin C or placebo, and 109 participants were included in the final analysis. Participants received Aptamin C at a dose of 36.057 mg/day or placebo for 4 weeks. The results showed significant increases in NK cell cytotoxicity after 2 and 4 weeks in the Aptamin C group. Additionally, serum levels of cytokines and cytotoxic granules associated with NK cell activity peaked 4 weeks after Aptamin C intake. Subgroup analysis showed that the enhancing effect of Aptamin C on NK cell activity was mainly observed in participants older than 40 years, whereas no significant effects were detected in participants aged <40 years. In the in vitro study, NK-92 cells treated with Aptamin C were compared with NK-92 cells treated with vitamin C. Aptamin C treatment enhanced proliferation, survival, cytotoxicity, and cytotoxic granule production in NK-92 cells compared with vitamin C treatment. These findings indicate that Aptamin C may effectively promote NK cell activation, particularly in middle-aged and older adults, and suggest its potential as an immunomodulatory supplement for supporting NK cell function.

## 1. Introduction

Natural Killer (NK cells are innate lymphocytes that constitute approximately 10–15% of the peripheral blood lymphoid population [1,2]. These lymphocytes are prominently distributed in immune-relevant tissues, such as the spleen, bone marrow, liver, lungs, and lymph nodes, reflecting their crucial role in the immune surveillance and defense mechanisms of the body [3].

NK cells are classified into distinct subsets based on the expression levels of CD56 and CD16. CD56^bright^CD16^dim^ NK cells, characterized by their predominant secretion of IFN-γ and other pro-inflammatory cytokines, play a key role in modulating immune responses and controlling tumor growth by inducing cancer cell dormancy and eliminating circulating tumor cells [4]. In contrast, CD56^dim^CD16^+^ NK cells exhibit potent cytotoxic activity mediated by perforin and granzyme B, which are pivotal for inducing apoptosis in the target cells [5,6].

NK cell activation is initiated by cytokines such as TNF-α, IL-2, IL-12, IL-15, and IL-18, which are released by dendritic cells and other immune cells during the early stages of inflammation [7,8]. These cytokines enhance NK cell cytolytic functions, including IFN-γ production, which improves their anti-tumor and antiviral responses [9]. The decline in NK cell activity associated with aging and various pathological conditions highlights the importance of maintaining their optimal function in immune surveillance and defense against cancer and infections such as COVID-19 [10,11,12,13]. Because this decline becomes more pronounced from middle age onward, NK-targeted interventions may be of greater benefit in older adults.

Various approaches for enhancing NK cell function have been investigated. For instance, various natural compounds, such as vitamins, glutamine, ginseng extract, and lectin, are particularly effective in NK cell activation [14]. Vitamin C, an essential antioxidant, has been extensively studied for its immunomodulatory effects, particularly its role in supporting NK cell activity [15,16,17]. Vitamin C protects NK cells from oxidative stress, promotes their proliferation, and enhances their cytotoxic potential against infected and malignant cells [14,18,19,20,21]. Despite these beneficial effects, the biological activity of vitamin C is limited by its chemical instability, particularly its susceptibility to oxidation under physiological and storage conditions. To address this limitation, stabilized vitamin C derivatives such as ascorbyl palmitate and magnesium ascorbyl phosphate have been developed [22,23]. Although these derivatives improve stability, some exhibit lower bioavailability than native vitamin C, which may reduce their physiological effectiveness in vivo [23,24]. An ideal approach, therefore, would preserve the biological activity of vitamin C and improve its stability without sacrificing bioavailability.

Aptamers are short, single-stranded nucleic acid molecules that have emerged as promising tools for targeted drug delivery because of their high specificity and minimal immunogenicity [25]. In this context, Aptamin C320 was developed as an aptamer–vitamin C conjugate designed to stabilize vitamin C while preserving, or even enhancing, its bioavailability and antioxidant efficacy [26]. This strategy may help overcome two major limitations of conventional approaches: the rapid oxidation of vitamin C and the reduced bioavailability observed with some of its stabilized derivatives. Recent studies have demonstrated the anti-inflammatory properties of Aptamin C320 in models of inflammation triggered by house dust mite extract [27], suggesting its therapeutic applications, including the potential enhancement of NK cell function [28,29].

This study was designed to evaluate whether oral administration of Aptamin C320 enhances NK cell activity in humans and to characterize its cellular effects using an in vitro NK cell model. We hypothesized that 4 weeks of Aptamin C320 intake would increase NK cell cytotoxicity compared with placebo. Accordingly, the primary efficacy endpoint of the human study was the change in NK cell cytotoxicity from baseline to Week 4, while serum cytokines and cytotoxic granules associated with NK cell activation were assessed as secondary outcomes. An age-based subgroup comparison was performed as an exploratory analysis to determine whether the response to Aptamin C320 differed by age. The in vitro experiments using NK-92 cells compared the effects of Aptamin C320 and vitamin C on NK cell proliferation, survival, cytotoxicity, and cytotoxic granule production. By extending our previous mechanistic findings [28] to a randomized, placebo-controlled human setting, the present study establishes the clinical relevance of Aptamin C320 for NK cell activation and reveals a previously unreported age-dependent effect.

## 2. Materials and Method

### 2.1. Cell Culture

NK-92, a human natural killer cell line, obtained from the American Type Culture Collection (ATCC, Manassas, VA, USA), was cultured in Alpha MEM medium (WELGENE, Kyungsan, Republic of Korea) supplemented with 12.5% heat-inactivated FBS and horse serum (Gibco, Grand Island, NY, USA), antibiotics (100 U/mL penicillin and 100 μg/mL streptomycin; WELGENE), 0.02 mM folic acid (Sigma, St. Louis, MO, USA), 0.2 mM myoinositol (Sigma), 0.1 mM 2-mercaptoethanol (Gibco), and 200 IU/mL recombinant IL-2 (Miltenyi Biotec, Bergisch Gladbach, Germany). K-562, a human myeloid leukemia cell line, was maintained in RPMI 1640 medium (WELGENE) containing 10% heat-inactivated FBS (Gibco) and antibiotics (100 U/mL penicillin and 100 μg/mL streptomycin; WELGENE). The cells were cultured at 37 °C in a humidified incubator with 5% CO_2_. Cell number and viability were assessed using the trypan blue exclusion assay.

### 2.2. Study Design, Participants, Randomization, and Intervention

This was a 4-week, randomized, double-blind, placebo-controlled trial carried out at three clinical sites: Severance Hospital (4-2022-0914), Seoul St. Mary’s Hospital of the Catholic University of Korea (KC22HODE0522), and Penta-heal Clinic (2022-0776-012). The study ran from 1 November 2022, when the first participant entered the trial, to 3 May 2023, when the last participant completed it, and enrolled 120 participants aged 19–75 years.

After providing written informed consent, candidates were screened against predefined inclusion and exclusion criteria and then randomly assigned to the Aptamin C or placebo group. The study population comprised generally healthy adults with normal immune function at enrollment, defined in part by a peripheral blood leukocyte count of 4000–8000 cells/μL at the screening visit. Individuals were excluded if they had a clinically significant acute or chronic illness, were taking medications or supplements that could affect immune function, had recently participated in another clinical trial, or had any other condition that, in the investigator’s judgment, could interfere with participation or outcome assessment.

Randomization and blinding were handled by an independent contract research organization (CRO). Eligible participants were allocated 1:1 to the Aptamin C or placebo group using a computer-generated, permuted-block sequence stratified by sex to balance the groups; age was not a stratification factor, so age-based subgroup analyses were performed after data collection as exploratory analyses to examine whether the response to Aptamin C differed by age group. The CRO held the randomization codes so that investigators could not anticipate the next participant’s assignment. The active product and placebo were identical in appearance, packaging, and labeling, each carrying a unique randomization code, and investigators dispensed only the product matching each participant’s number. Blinding was maintained for participants, investigators, clinical staff, laboratory analysts, outcome assessors, and statisticians throughout, and the allocation codes were not released until database lock and completion of the final analysis.

The daily dose of Aptamin C was set by the approved manufacturing specification of the previously developed formulation rather than by a formal human-equivalent-dose conversion from animal data. Under that specification, each 500 mg tablet contained 36.057 mg of Aptamin C, defined as the vitamin C–DNA aptamer complex excluding excipients; participants in the Aptamin C group therefore took one tablet daily, equivalent to 36.057 mg/day. Participants took the assigned product once daily, 30 min after breakfast, for the full 4-week intervention. Blood samples were collected at baseline before administration of the study product and again at Weeks 2 and 4 during the intervention period. The placebo tablet was prepared by replacing the Aptamin C complex with citric acid while keeping all other excipients identical to those in the active tablet. General nutritional and lifestyle variables were recorded during the study, but dietary vitamin C intake was not measured with a food diary or food frequency questionnaire. Participants were asked not to start any new dietary supplements, including those containing vitamin C, during the study, and supplement use was monitored.

### 2.3. Participant Disposition and Analysis Populations

Of the 149 individuals screened, 29 failed screening, and the remaining 120 eligible participants were randomized to the Aptamin C group (*n* = 61) or the placebo group (*n* = 59). All randomized participants received the investigational product at least once and were therefore included in the Safety Set. Five participants were excluded from the Full Analysis Set because of an eligibility-criterion violation or sample loss after Visit 2, leaving 115 participants (57 in the Aptamin C group and 58 in the placebo group). Following a blind review meeting on 20 September 2023, six further participants were excluded from the Per-Protocol Set for protocol-related reasons—compliance below 70%, use of prohibited concomitant medication, failure to return the investigational product, or an adverse event/serious adverse event. The Per-Protocol Set thus comprised 109 participants (54 in the Aptamin C group and 55 in the placebo group) and served as the primary efficacy analysis population.

### 2.4. Human NK Cell Isolation

Peripheral blood mononuclear cells (PBMCs) were isolated from 30 mL peripheral blood samples by density-gradient centrifugation over Ficoll-Paque (Sigma-Aldrich). These samples were drawn from the study participants described in Section 2.3. RBCs were lysed using a 1× RBC lysis solution (BioLegend, San Diego, CA, USA). NK cells were isolated from PBMCs using an NK Cell Isolation Kit (Miltenyi Biotec, Bergisch Gladbach, Germany). The cells were labeled with an NK cell biotin antibody and NK cell microbead cocktail, followed by negative selection using an autoMACS pro separator. The purity of the NK cells was confirmed by staining with FITC-conjugated anti-human CD3 (BioLegend) and PE-conjugated anti-human CD56 (BD Biosciences, San Jose, CA, USA), resulting in a purity of 94% for use in cytotoxicity assays.

### 2.5. Cytotoxicity Assay

NK cell cytotoxicity was assessed using the PKH-26/7-AAD assay. NK cells were co-cultured with PKH-26-labeled K-562 cells at ratios of 10:1 and 20:1 for 4 h at 37 °C with 5% CO_2_. After incubation, cells were stained with 7-AAD (BD Bioscience) at a concentration of 2 μL per 1 × 10^5^ cells to detect dead cells and analyzed by flow cytometry using FlowJo software version 10.8.1 (BD Bioscience). Flow cytometry was performed using a sequential gating strategy. (1) A population of intact cells was selected based on the forward scatter area (FSC-A) versus the side scatter area (SSC-A) to exclude debris. (2) Single cells were gated using FSC-A versus forward scatter height (FSC-H) to remove doublets. (3) The target cell population was identified as PKH-26^+^ using unstained, PKH-26 single-stained, and effector-only control samples to define positive and negative boundaries. (4) Within the PKH-26^+^ gate, cells positive for 7-AAD were considered dead. Cytotoxicity was calculated as the percentage of 7-AAD^+^PKH-26^+^ cells among total PKH-26^+^ target cells using the following formula:

$$\%\;Cytotoxicity=\frac{7AAD^{+}\;{PKH26}^{+}\;cells}{{total\;PKH26}^{+}\;cells}\times 100$$

PKH-26-negative cells, corresponding to NK effector cells, were excluded from the cytotoxicity analysis. Representative flow cytometry plots are shown in the figures, whereas quantitative bar graphs summarize the results from independent repeated experiments. Therefore, minor differences between representative flow cytometry plots and the values presented in the bar graphs may be observed.

### 2.6. LDH Assay

To evaluate cytotoxicity, NK cells were co-cultured with K-562 cells at ratios of 10:1 and 20:1 for 4 h at 37 °C with 5% CO_2_. Effector spontaneous release controls (NK cells alone), target spontaneous release controls (K-562 cells with assay buffer), and target maximum release controls (K-562 cells with lysis buffer) were included in each experiment. Absorbance was measured at 490 nm using SpectraMax iD3 (Molecular Devices, San Jose, CA, USA), and results were normalized using SoftMax Pro software version 7.0 (Molecular Devices). Cytotoxicity was calculated using the following formula:

$$Cytotoxicity\left(\%\right)=\frac{\left(Experimental\;release-Effector\;spontaneous\;release-Target\;spontaneous\;release\right)}{\left(Target\;maximum\;release-Target\;spontaneous\;release\right)}\times 100$$

This normalization accounts for spontaneous LDH release from both effector and target cells, thereby minimizing the influence of NK cell death on the calculated cytotoxicity values.

### 2.7. Multiplex Cytometric Bead Assay

Serum samples were collected from study participants at 0, 2, and 4 weeks after intake of either Aptamin C or placebo. Serum levels of IL-2, IFN-γ, TNF-α, granulysin, granzyme A, granzyme B, and perforin were evaluated using the LEGENDplex Human CD8/NK Panel (BioLegend). For the in vitro experiments, supernatants were collected from NK-92 cells treated with Aptamin C for 48 h and co-cultured with K-562 cells for 4 h. Granzyme B and perforin levels in the culture supernatants were analyzed using the same assay. Samples were prepared according to the manufacturer’s instructions and analyzed using flow cytometry. Data analysis was performed using LEGENDplex™ Data Analysis Software Suite (Qognit Inc., San Carlos, CA, USA).

### 2.8. CCK-8 Assay

To determine the appropriate IL-2 concentration for subsequent NK-92 cell experiments, NK-92 cells were seeded in a 96-well plate and treated with varying concentrations of IL-2. After incubation for 24 or 48 h, 10% of the total volume of the CCK-8 reagent (Cell proliferation and cytotoxicity assay kit, EZ-Cytox; DOGEN, Seoul, Republic of Korea) was added to each well. The absorbance at 450 nm was measured using a SpectraMax iD3. The results were normalized using Softmax Pro software version 7.0.

### 2.9. Alamar Blue Assay

For proliferation analysis, NK-92 cells were treated with IL-2 and Aptamin C for 48 h. Cell morphology was examined under a microscope at 40× magnification after 48 h of treatment (scale bar = 750 μm). Cell proliferation was subsequently assessed using the Alamar Blue assay. Briefly,. Alamar Blue reagent (Serotec, Kidlington, UK) was added to each well at 10% of the total well volume. Following a 6 h incubation at 37 °C without exposure to light, the absorbance was measured at 570 nm (reduced form) and 600 nm (oxidized form) using SpectraMax iD3. The results were normalized using Softmax Pro software, and proliferation was calculated using the reduction (%) formula:

$$Reduction\left(\%\right)=Absorbance\frac{Reduced(sample-background)-Oxidized(sample-background)}{Reducded(control-background)-Oxidized(control-background)}\times 100$$

### 2.10. Statistical Analysis

Experimental data are presented as the mean ± SD or SEM. Differences among three or more groups were assessed by one-way ANOVA followed by Tukey’s post hoc test for multiple comparisons, and differences between two groups by an unpaired two-tailed Student’s *t*-test; two-way ANOVA was used to evaluate the effects of treatment group and time point. These analyses were performed in GraphPad Prism version 8.0.2 (GraphPad Software, La Jolla, CA, USA), with statistical significance set at *p* < 0.05. Data from the human application test were analyzed using SAS version 9.4 (SAS Institute, Cary, NC, USA), with all tests two-sided at α = 0.05; *p*-values are reported to four decimal places and descriptive statistics to two. Three analysis populations were defined: the Safety Set, the Full Analysis Set (FAS), and the Per-Protocol Set (PPS). Efficacy, demographic, and nutritional variables were analyzed primarily on the PPS—with the FAS as a supportive analysis for efficacy—whereas safety variables were analyzed on the Safety Set. Missing efficacy values were imputed by last observation carried forward (LOCF) but were not replaced with baseline values, and non-efficacy variables were analyzed as observed. Continuous variables are expressed as mean ± SD, and normality was tested by the Shapiro–Wilk method. Between-group comparisons (test vs. control) used a two-sample *t*-test when both groups were normally distributed and the Wilcoxon rank-sum test otherwise, while within-group changes (baseline vs. endpoint) used a paired *t*-test or, where normality was not met, the Wilcoxon signed-rank test. Categorical variables, reported as frequencies and percentages, were compared by the chi-square test, or Fisher’s exact test when expected cell counts were small. The primary endpoint, NK cell activity, was analyzed as the change from baseline compared between the test and control groups using the two-sample *t*-test or Wilcoxon rank-sum test according to normality. In an exploratory age-stratified analysis, the change from baseline in NK cell function at Weeks 2 and 4 was analyzed by ANCOVA, with treatment group, age group (<40 vs. ≥40 years), and the treatment-by-age-group interaction as fixed effects and baseline NK cell function as a covariate; the treatment-by-age-group interaction tested whether the effect of Aptamin C differed by age group.

## 3. Results

### 3.1. Participant Disposition and Baseline Characteristics

**Figure 1:**
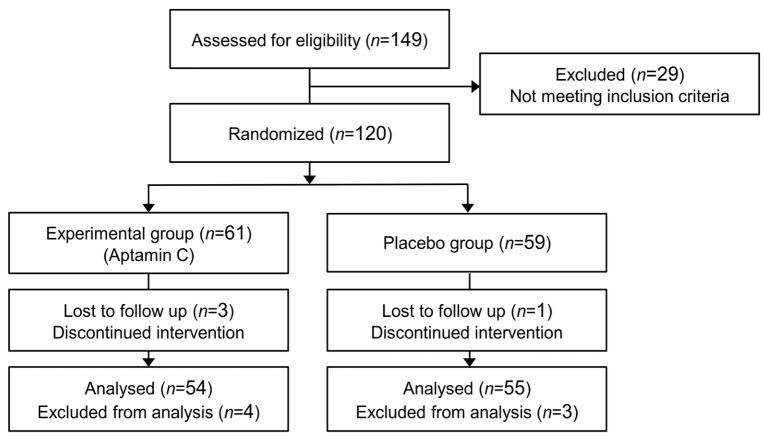
Designs of the in vivo study.

The study included participants whose baseline characteristics were generally comparable between the placebo and Aptamin C groups (Table 1 and Figure 1). The placebo group consisted of 55 individuals with a mean age of 45.76 ± 11.63 years, of whom 13 (23.64%) were male. The Aptamin C group consisted of 54 individuals with a mean age of 44.33 ± 12.19 years, with 18 (33.33%) identified as male. Among the 149 participants initially screened, 29 were excluded because of unmet criteria or consent withdrawal. The remaining 120 participants were randomized into two groups: 61 and 59 in the Aptamin C group and placebo group, respectively. After dropping out, 115 participants completed the study: 57 in the Aptamin C group and 58 in the placebo group. The per-protocol set included 54 participants in the Aptamin C group and 55 in the placebo group. For an exploratory age-based analysis using a 40-year cutoff, the placebo group comprised 41 participants aged ≥40 years and 14 aged <40 years, and the Aptamin C group comprised 36 aged ≥40 years and 18 aged <40 years. Differences by age group were assessed by ANCOVA with baseline NK cell function as a covariate. The study was implemented in three hospitals with a double-blind design, and participants received either Aptamin C (36.057 mg/day) or placebo for 4 weeks. Blood was collected at baseline, before administration of the study product, and again at weeks 2 and 4 during the intervention period. NK cells and serum isolated from these blood samples were used to assess NK cell activity by measuring NK cell cytotoxicity as well as granule and cytokine levels.

### 3.2. Aptamin C Supplementation Enhances NK Cell Cytotoxicity

**Figure 2:**
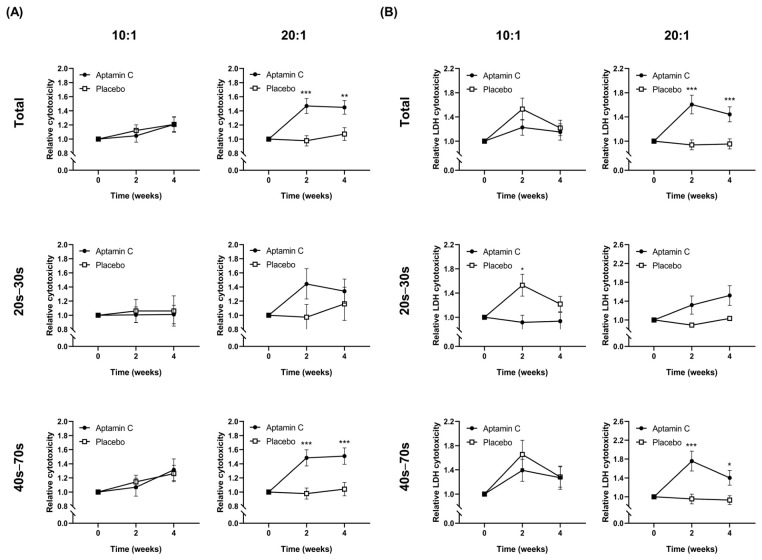
The Aptamin C group (*n* = 54) and placebo group (*n* = 55) were administered Aptamin C (36.057 mg/day) and placebo, respectively, for 4 weeks. (**A**) For flow cytometric analysis, K-562 cells were labeled with PKH-26 to distinguish them from NK cells. After co-culture with K-562 cells for 4 h, 7-AAD staining was performed to detect dead cells. (**B**) The co-culture supernatant and cells were used for the LDH assay. Cell damage was detected using the LDH assay. The cytotoxicity assays were conducted at effector-to-target (E/T) ratios of 10:1 and 20:1. Data values are presented as means ± SEM. Statistical significance is denoted by * *p* < 0.05, ** *p* < 0.01, and *** *p* < 0.001.

To evaluate the cytotoxic ability of NK cells, NK cells isolated from blood samples were co-cultured with K-562 at effector/target (E/T) ratios of 10:1 and 20:1 for 4 h. PKH-26/7-AAD assay was performed using K-562-labeled with PKH-26. In the overall population, at a 10:1 ratio, the PKH-26/7-AAD assay results showed no significant difference between the placebo and Aptamin C groups. However, at a 20:1 ratio, the results after 2 and 4 weeks demonstrated that the Aptamin C group had significantly increased cytotoxicity compared to the placebo group (Figure 2A). When the results were analyzed by age group, in individuals aged <40 years, no significant difference in NK cell cytotoxicity was observed between the Aptamin C and placebo groups in the PKH-26/7-AAD assay at both the 10:1 and 20:1 effector-to-target ratios. However, in participants aged 40 years and older, NK cell cytotoxicity significantly increased after 2 and 4 weeks of Aptamin C consumption compared to the placebo group at a 20:1 effector-to-target ratio (Figure 2A). Additionally, an LDH assay was conducted using the co-cultured cells and supernatants. Similar to the PKH-26/7-AAD assay, in all age groups, the LDH assay results showed no significant differences between the placebo and Aptamin C groups at the 10:1 ratio. However, at the 20:1 ratio, the Aptamin C group exhibited significantly higher cytotoxicity than the placebo group (Figure 2B). The LDH assay results for different age groups were consistent with these findings, showing no significant differences in the <40 age group but a marked increase in NK cell cytotoxicity for ages 40 and above after 2 and 4 weeks of Aptamin C consumption compared to the placebo group (Figure 2B). These findings suggest that the increase in NK cell cytotoxicity due to Aptamin C consumption becomes more noticeable as age increases. To formally test whether this age-related difference was statistically supported, a baseline-adjusted ANCOVA including treatment group, age group, and their interaction (treatment × age group) was fitted, with the corresponding baseline value as a covariate. The treatment × age-group interaction was significant at Week 2 in both the 7-AAD- and LDH-based assays (*p* = 0.0036 and *p* = 0.0019, respectively), but not at Week 4 in either assay (*p* = 0.2052 and *p* = 0.1840, respectively), indicating that the age-related difference in the treatment effect was more pronounced at Week 2 than at Week 4. Notably, the LDH assay results showed that NK cell activity was greater in the LDH assay results after 4 weeks of Aptamin C administration than in the PKH-26/7-AAD assay results (Figure 2B). This discrepancy likely arises because the LDH assay reflects the results of both dead and dying cells, whereas the PKH-26/7-AAD assay counts only dead cells.

### 3.3. Aptamin C Increases the Levels of Cytokines and Cytotoxic Granules in Serum

**Figure 3:**
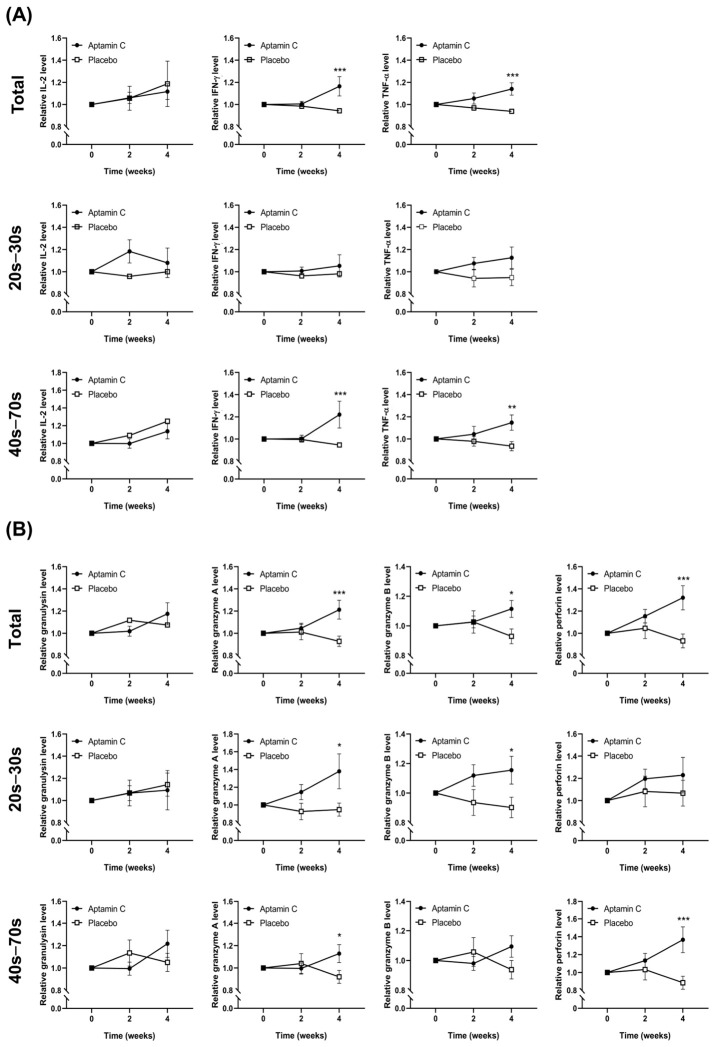
Effect of Aptamin C on the release profile of cytokines and cytotoxic granules. The study included an Aptamin C group (*n* = 54) and a placebo group (*n* = 55) administered Aptamin C and placebo, respectively, for 4 weeks. Effect of Aptamin C on serum cytokine and granule levels at weeks 0, 2, and 4. (**A**) The concentrations of IL-2, IFN-γ, and TNF-α in serum were assayed using multiplex cytometric bead assay, following the manufacturer’s instructions. (**B**) Serum granule levels of granulysin, granzyme A, granzyme B, and perforin were evaluated. Results are presented as means ± SEM. Statistical significance is denoted by * *p* < 0.05, ** *p* < 0.01, and *** *p* < 0.001.

To measure the effect of Aptamin C on NK cell activity, cytokine and granule levels in serum were compared between the Aptamin C and placebo groups using a multiplex cytometric bead assay. In all age groups, the IL-2 levels were not significantly different between the two groups. However, IFN-γ and TNF-α levels significantly increased in the Aptamin C group compared to the placebo group after 4 weeks of supplementation. When the results were analyzed by age group, in participants aged <40 years, no significant differences were observed between the Aptamin C and placebo groups in the levels of IL-2, TNF-α, and IFN-γ. However, in participants aged 40 years and older, the levels of TNF-α and IFN-γ (but not of IL-2) significantly increased after 4 weeks of Aptamin C consumption compared to the placebo group (Figure 3A). Therefore, Aptamin C induced an increase in NK cell activity and serum cytokine levels, indicating that immune function differs with age. In addition, the serum levels of cytotoxicity-related granules were measured for the Aptamin C and placebo groups. Granulysin levels were not significantly different between the two age groups. However, granzyme A, granzyme B, and perforin levels were significantly higher in the Aptamin C group than in the placebo group after 4 weeks of supplementation. Individuals aged <40 years in the Aptamin C and placebo groups showed no significant differences in the levels of granulysin and perforin. However, the levels of granzyme A and granzyme B significantly increased after 4 weeks of Aptamin C consumption compared to the placebo group. In participants aged 40 years and older, the levels of granzyme A and perforin (but not of granulysin and granzyme B) significantly increased after 4 weeks of Aptamin C consumption compared to those in the placebo group (Figure 3B). Consequently, Aptamin C-treated NK cells showed an age-dependent increase in sensitivity to cancer cells, with increased cytotoxic activity mediated by the secretion of cytokines and cytotoxic granules. Our findings show that Aptamin C enhances immune responses, particularly in older adults, by increasing the levels of key cytokines and granules in the serum. Using the same ANCOVA model, significant treatment × age-group interactions were observed at Week 4 for IFN-γ, TNF-α, granzyme B, and perforin (*p* = 0.0157, 0.0091, 0.0330, and 0.0001, respectively), indicating that the effect of Aptamin C on these mediators differed by age group. Accordingly, evidence of treatment-effect modification within the age subgroups was considered only for outcomes in which the interaction term reached statistical significance.

### 3.4. Safety and Tolerability

Safety was monitored throughout the trial through adverse event reporting and clinical laboratory testing, including hematology, blood chemistry, and urinalysis. Over the 4-week intervention, the Aptamin C group showed no clinically significant laboratory abnormalities, and no serious adverse events were attributed to Aptamin C. Aptamin C was therefore well tolerated at a daily dose of 36.057 mg.

### 3.5. Aptamin C Enhances Survival and Proliferation of NK-92

**Figure 4:**
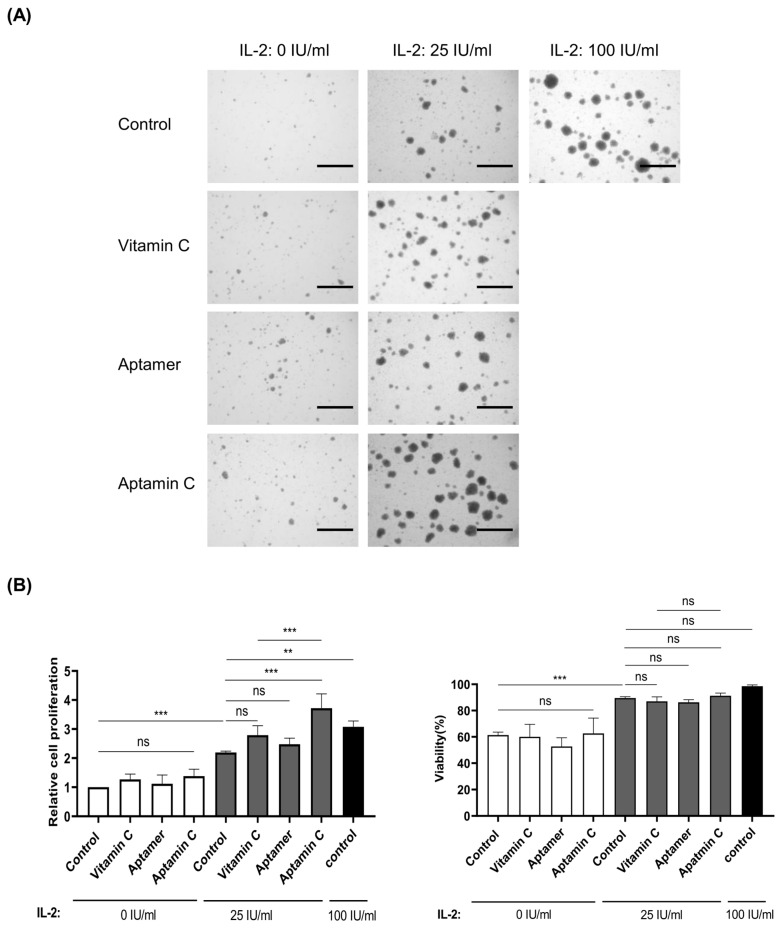
Effect of Aptamin C on survival and proliferation in NK-92. NK-92 cells were seeded in a 96-well plate at a density of 1 × 10^4^ cells per well and supplemented with 0, 25, or 100 IU/mL of IL-2 for 48 h. NK-92 cells were treated with vitamin C, aptamer, or Aptamin C for 48 h in the presence or absence of IL-2 (25 IU/mL). (**A**) Morphological assessment of NK-92 cells was performed after 48 h under a microscope at 40× magnification. Scale bar: 750 μm. (**B**) Alamar Blue assay was performed to evaluate NK-92 proliferation, and cell number and viability were measured using the trypan blue dye exclusion assay. Data represent three independent experiments with values presented as means ± SD. ** *p* < 0.01, *** *p* < 0.001, ns: not significant.

To investigate whether Aptamin C increases NK-92 cell proliferation and survival, microscopic examination of cell morphology and the Alamar blue assay were performed. NK-92 cells not treated with IL-2 displayed minimal cell numbers and failed to form colonies. In contrast, NK-92 cells treated with IL-2 (25 IU/mL, Figure S1) displayed increased cell and colony formation compared with NK-92 cells without IL-2. When co-treated with IL-2 and Aptamin C, colony formation was similar to that in the positive control (100 IU/mL of IL-2). The results showed a notable increase in colony formation within the Aptamin C-treated groups (20 μg/mL, Figure S2), suggesting NK-92 activation (Figure 4A). Without IL-2, no significant differences in cell numbers were observed between the Aptamin C and control groups, with a notable decrease in viability to 60%. NK-92 cells treated with IL-2 showed increased cell numbers compared to NK-92 cells not treated with IL-2. However, when co-treated with IL-2 and Aptamin C, the cell numbers were comparable to those of the positive control (100 IU/mL of IL-2) (Figure 4B).

### 3.6. Aptamin C Enhances NK-92 Cytotoxicity Against K562 and Increases Cytotoxic Granule Release 

**Figure 5:**
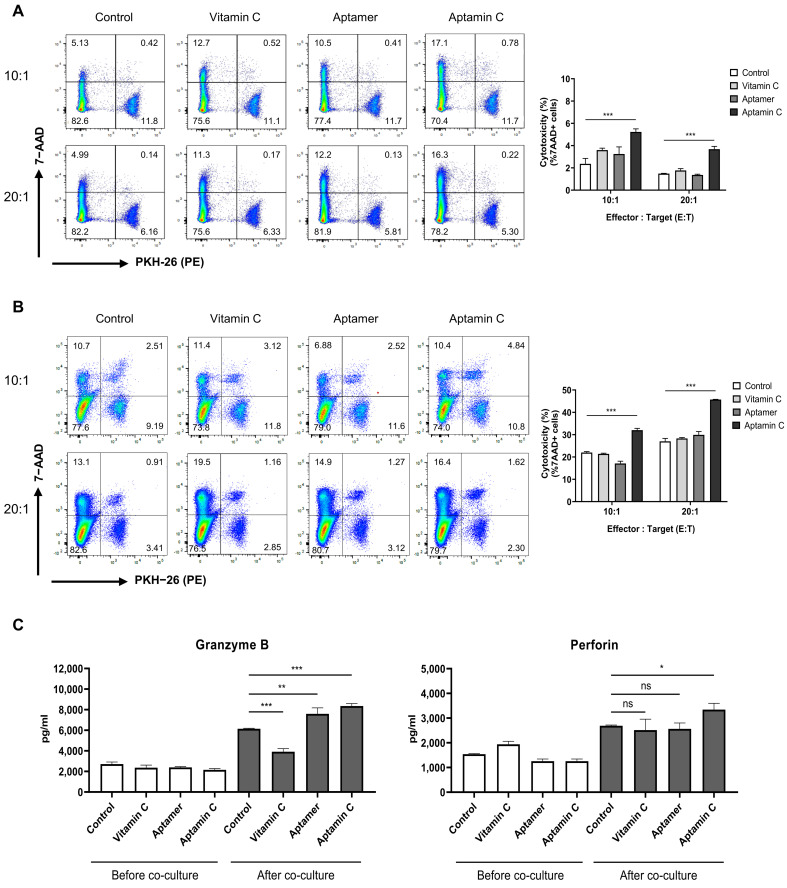
Effect of Aptamin C on cytotoxicity and release profile of cytotoxic granules in NK-92. (**A**) NK-92 cells cultured with IL-2 (25 IU/mL) were treated with vitamin C, aptamer, or Aptamin C for 48 h. (**B**) NK-92 cells were cultured in the presence of IL-2 (25 IU/mL) and treated with vitamin C, aptamer, or Aptamin C for 48 h. K-562 cells were labeled with PKH-26 to distinguish them from NK-92 cells. After 4 h of co-culture with K-562 cells, dead target cells were identified by 7-AAD staining. NK cell cytotoxicity was assessed at effector-to-target (E/T) ratios of 10:1 and 20:1 as the percentage of 7-AAD-positive K-562 cells. (**C**) NK-92 cells cultured with IL-2 were incubated overnight, and culture supernatants were collected before and after co-culture with K-562 cells. Granzyme B and perforin concentrations were measured using a multiplex cytometric bead assay. Data are presented as means ± SD. * *p* < 0.05, ** *p* < 0.01, *** *p* < 0.001, ns: not significant.

Through in vivo experiments, it was confirmed that Aptamin C increased cytotoxicity and the levels of cytokines and granules in peripheral blood natural killer cells. The cytotoxicity was assessed to determine whether they yielded similar outcomes. The results showed that although NK-92 cells untreated with IL-2 displayed lower cytotoxicity than IL-2-treated NK-92 cells, the Aptamin C-treated group exhibited higher cytotoxicity than the other groups (Figure 5A). NK-92 cells treated with IL-2 (25 IU/mL) showed higher cytotoxicity in the Aptamin C-treated group than in other groups (Figure 5B). To examine whether this increased cytotoxicity involved the release of granzyme B and perforin cytotoxic granules, supernatants from the cytotoxicity experiments at an effector-to-target (E/T) ratio of 20:1 were analyzed. The results showed that Aptamin C-treated NK-92 cells with IL-2 exhibited increased levels of granzyme B and perforin when co-cultured with target cells (Figure 5C). Therefore, Aptamin C enhanced NK cell cytotoxicity and the secretion of granzyme B and perforin.

## 4. Discussion

There is sufficient evidence demonstrating the antioxidant functions of vitamin C in the body, which extend beyond reducing reactive oxygen species by donating electrons while enhancing NK cell function [13,30,31]. Aptamin C, by utilizing aptamers, enhances the stability of vitamin C when exposed to external conditions [26]. In this study, the in vivo results suggested that the consumption of Aptamin C resulted in increased NK cell cytotoxicity and serum levels of cytotoxicity-related granules and cytokines compared to the placebo group (Figure 2 and Figure 3). Specifically, only TNF-α and IFN-γ levels increased, suggesting that IL-2 may not directly contribute to the mechanism by which Aptamin C enhances NK cell activation. Instead, IL-2 activates NK cells upon exposure, rather than being primarily produced during NK cell activation [32,33,34]. We observed an increase in granzyme A, granzyme B, and perforin levels, which were associated with cytotoxicity. Granzyme A targets nuclear proteins, particularly histones, causing DNA damage via a caspase-independent pathway [35,36]. Granzyme B, which enters the cytoplasm via perforin, induces apoptosis by cleaving and activating the Bid protein, thereby inducing mitochondrial damage via a caspase-dependent pathway [37,38]. These results suggested that Aptamin C enhanced NK cell function by boosting cytotoxicity via granule-mediated mechanisms.

In healthy participants without cancer, Aptamin C intake can increase serum cytokines and granules, suggesting the activation of other immune cells to protect against potential threats [39]. Additional in vitro experiments were conducted to address this issue. The results showed that Aptamin C itself did not elevate granules levels in the absence of K562 target cells; however, it specifically increased granule levels in their presence (Figure 5). Nevertheless, the possibility that increased cytokine and granule levels in the serum are induced by other immune cells in healthy participants cannot be excluded. Studies using Gulo−/− mice lack functional L-gulonolactone oxidase and, like humans, cannot synthesize vitamin C endogenously [40], investigated NK cell receptor expression, cytotoxicity, signaling pathways, and other immune cell responses to Aptamin C [28]. These results provide additional evidence of the immunomodulatory effects of Aptamin C.

As individuals age, their NK cell functionality tends to diminish [41]. In our study, we observed that the cytotoxic capacity of NK cells did not improve with Aptamin C consumption in participants aged <40 years. However, in participants aged 40 years and older, Aptamin C significantly enhanced NK cell cytotoxicity. Additionally, when comparing serum cytokine and granule levels between the placebo and Aptamin C groups, no significant differences were found in the <40 age group, except for granzyme B. Granzyme B, which primarily relies on perforin to enter target cells [37,42], did not show a corresponding increase in perforin levels, suggesting that the lack of perforin augmentation may explain the absence of significant cytotoxicity improvements in this age group. In contrast, participants aged 40 years and older demonstrated increased cytotoxicity and elevated levels of NK cell-related cytokines and granules in the serum following Aptamin C consumption. These results imply that while Aptamin C may not enhance NK cell function in healthy young adults, it has a significant effect on individuals aged over 40 years, whose immune function may be compromised compared to younger individuals. The increase in NK cell cytotoxicity following Aptamin C intake appeared more evident in participants aged ≥40 years than in younger participants. This subgroup analysis was exploratory, as the study was neither designed nor powered to assess age-dependent effects; therefore, the observed difference between age groups should be interpreted with caution. Larger studies with predefined age stratification and formal age-by-treatment interaction testing are needed to confirm whether Aptamin C shows differential effects according to age. These findings suggest that Aptamin C may have value as a dietary supplement for supporting immune function in populations at risk of diminished NK cell activity due to aging. The clinical relevance of increased NK cell cytotoxicity in healthy adults should be interpreted with caution. NK cells play an important role in immune surveillance against virus-infected and malignant cells, and reduced NK cell activity has been associated with aging and impaired immune defense. In this context, the increase in NK cell cytotoxicity observed after Aptamin C intake may indicate improved innate immune responsiveness, particularly in middle-aged and older adults. However, the present study did not evaluate clinical outcomes such as the incidence or severity of infections, cancer-related outcomes, or responses to immunotherapy. Therefore, the observed immunological changes should not be interpreted as direct evidence of disease prevention or therapeutic benefit. Rather, these findings suggest that Aptamin C may support NK cell function, and future long-term studies in populations with reduced NK cell activity or clinically relevant immune challenges are needed to determine whether this effect translates into meaningful health benefits.

Because the randomized human study did not include a vitamin C comparator arm, the direct comparison between Aptamin C and vitamin C was limited to the in vitro NK-92 cell experiments. Aptamin C promoted NK-92 colony formation, improved NK cell survival, and significantly increased NK-92 cell proliferation compared with vitamin C (Figure 4B). These improvements in colony formation suggest an improved activation of signaling between cells, potentially promoting the activation and differentiation of effector lymphocytes. These findings align with those of previous research, indicating that a stable form of vitamin C can enhance immune cell function [43]. Additionally, Aptamin C enhanced NK-92 cytotoxicity against K-562 cells and increased the secretion of granzyme B and perforin compared to vitamin C (Figure 5C). It should be noted that the direct comparison between Aptamin C and vitamin C was performed only in the in vitro NK-92 cell system. The randomized human study evaluated Aptamin C against placebo and therefore does not allow a direct clinical comparison between Aptamin C and vitamin C. In aqueous solutions, Aptamin C oxidizes more slowly than vitamin C, resulting in a longer half-life [26]. Unlike other vitamin derivatives, Aptamin C ensures efficient vitamin C absorption in cells by separating the aptamer from Aptamin C under physiological conditions [44]. This property likely contributed to the observed enhancement of NK cell activity, as vitamin C stability is crucial for its immunomodulatory functions. The improved stability and bioavailability of Aptamin C align with the literature findings, suggesting that stabilized vitamin C forms are more effective.

Taken together, the in vitro NK-92 cell findings suggest that Aptamin C may enhance NK cell activation-related parameters more effectively than vitamin C alone. However, because the randomized human study included only Aptamin C and placebo groups, this comparison should be interpreted as limited to the in vitro findings and not as evidence of clinical superiority over vitamin C. This can have implications for NK cell-based immunotherapy, which is increasingly recognized for its safer profile compared to T cell-based therapies that pose risks such as graft-versus-host disease [45]. Current research has focused on refining ex vivo priming strategies to enhance NK cell therapy [46]. Although cytokine-based expansion promotes cell growth and enhances functionality, it requires periodic cytokine injections, leading to high costs and ‘cytokine addiction’ in NK cells [45,46,47]. Despite its excellent efficacy, IL-2 can lead to complications, such as abnormal immune cell activation and autoimmune risks [47,48,49]. To address these challenges, a cytokine cocktail, including IL-12, IL-15, and IL-18, has been developed; however, it remains costly [49,50,51]. Therefore, exploring natural substances for safer and more cost-effective NK cell activation is crucial. The addition of Aptamin C NK-92 cells cultured with one-quarter of the IL-2 concentration used in the positive-control condition produced effects comparable to those of the full IL-2 condition, enhancing colony formation, proliferation, cytotoxicity, and granule levels (Figure 4 and Figure 5). These in vitro findings suggest that Aptamin C may have potential as an adjunctive strategy for supporting NK cell activation under reduced IL-2 conditions. Further studies are needed to determine whether this approach can be applied to NK cell-based immunotherapy.

Another limitation of this study is that baseline plasma vitamin C concentrations and quantitative dietary vitamin C intake were not assessed. Although participants were instructed not to initiate new vitamin C-containing supplements during the study period, the potential influence of baseline vitamin C status or habitual dietary vitamin C intake on NK cell activity cannot be completely excluded. Future studies should include baseline plasma vitamin C measurements and detailed dietary assessments to better control for this potential confounder.

## 5. Conclusions

These results provide evidence that Aptamin C supplementation enhances NK cell activity, consistent with the improved stability of vitamin C [26]. In the in vitro NK-92 model, Aptamin C enhanced NK cell function more effectively than vitamin C, and low-dose IL-2 combined with Aptamin C further increased NK cell activity. Aptamin C therefore emerges as a promising candidate for augmenting NK cell function and a potential priming reagent in NK cell therapy.

## Figures and Tables

**Table 1:** Demographic characteristics of participants.

		Placebo *n* = 55	Aptamin C *n* = 54
Gender	Male	13 (23.64)	18 (33.33)
	Female	42 (76.36)	36 (66.67)
	*p*-value *		0.2619
Age (y)	Mean ± SD	45.76 ± 11.63	44.33 ± 12.19
	*p*-value *		0.5320
BMI (kg/m^2^)	Mean ± SD	23.05 ± 2.97	23.18 ± 3.28
	*p*-value *		0.8255
Weight (kg)	Mean ± SD	61.59 ± 12.06	63.83 ± 12.31
	*p*-value *		0.3188
Family history of Immune disorder	Yes	3 (5.45)	0 (0.00)
	No	52 (94.55)	54 (100.00)
	*p*-value *		0.2431

Data are represented as number (%) or mean ± SD, as appropriate. * Comparison between the Placebo and Aptamin C groups using Student’s *t*-test.

## Data Availability

The data presented in this study are available on request from the corresponding author. The data are not publicly available due to privacy and ethical restrictions related to participant information.

## Supplementary Materials

The following supporting information can be downloaded at https://www.mdpi.com/article/10.3390/antiox15070796/s1, Figure S1: Effect of IL-2 concentration on NK-92 proliferation and viability; Figure S2: Effects of vitamin C, aptamer, and Aptamin C on NK-92 viability.

## Abbreviations

NK	Natural killer
LOCF	last observation carried forward
PBMC	Peripheral blood mononuclear cell
PPS	Per-protocol set
FSC-A	Forward scatter area
SSC-A	Side scatter area
FAS	Full analysis set
FSC-H	Forward scatter height
