# Supplementary material for: Effect of Aptamin C on NK Cell Activity and Cytotoxicity: A Randomized Placebo-Controlled Trial and In Vitro Comparison with Vitamin C
Source: Antioxidants (Basel). 2026 Jun 25;15(7):796. doi: 10.3390/antiox15070796 (PMC13405745; doi:10.3390/antiox15070796)
Supplement: Supplementary file 1 [file antioxidants-15-00796-s001.zip › antioxidants-4316790-supplementary.pdf]

antioxidants

MDPI

Article

Effect of Aptamin C on NK Cell Activity and Cytotoxicity: A Randomized Placebo-Controlled Trial and In Vitro Comparison with Vitamin C

Hyovin Ahn [1,2], June Lee [3], Jeong-Ho Park [3], Jae Sang Barn [4], Yejin Kim [1,2,5,*] and Jae Seung Kang [1,2,5,6,7,*]

[1] Laboratory of Vitamin C and Antioxidant Immunology, Department of Anatomy and Cell Biology, Seoul National University College of Medicine, Seoul 03080, Republic of Korea; jahb1220@snu.ac.kr
[2] Institute of Allergy and Clinical Immunology, Medical Research Center, Seoul National University, Seoul 08826, Republic of Korea
[3] Nexmos, Inc., Yongin-si 16827, Republic of Korea; ratury87@gmail.com (J.L.); joyjura80@gmail.com (J.-H.P.)
[4] BABOBAGI Plastic Surgery, 517 Nonhyeon-ro, Gangnam-gu, Seoul 06129, Republic of Korea; barn8088@naver.com
[5] Department of Research and Development, N Therapeutics Co., Ltd., Seoul 08813, Republic of Korea
[6] Artificial Intelligence Institute, Seoul National University, Seoul 08826, Republic of Korea
[7] Department of Applied Bioengineering, Graduate School of Convergence Science and Technology, Seoul National University, Seoul 08826, Republic of Korea
* Correspondence: bbambaya921@snu.ac.kr (Y.K.); genius29@snu.ac.kr (J.S.K.); Tel.: +82-2-740-8132 (J.S.K.)

Supplementary Materials

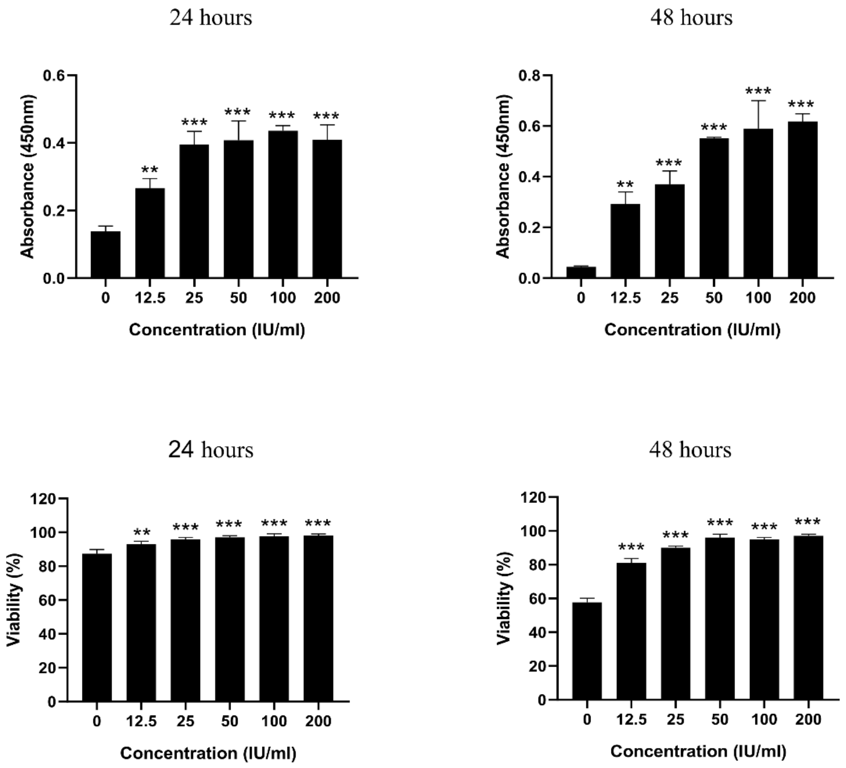

Figure S1. Effect of IL-2 concentration on NK-92 cells proliferation and viability. NK-92 cells were seeded in a 96-well plate at a density of 1 × 10^4 cells per well and supplemented with 12.5, 25, 50, 100, or 200 IU/mL of IL-2 for 24 and 48 h. The CCK-8 assay was

performed to evaluate NK-92 cells proliferation, and viability was assessed using trypan blue dye exclusion assay. Data represent three independent experiments with values presented as means ± SD. ** p<0.01, *** p<0.001.

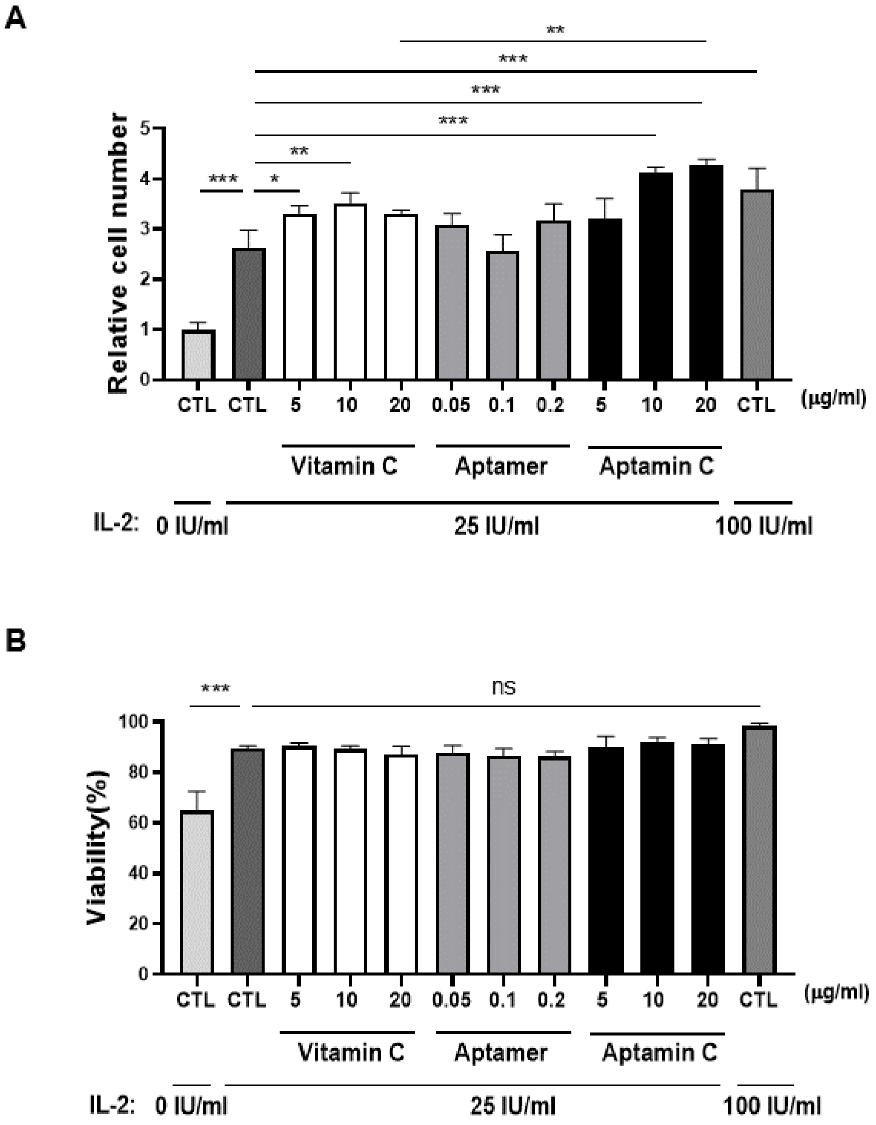

Figure S2. Effects of vitamin C, aptamer, and Aptamin C on NK-92 cells viability. NK-92 cells were seeded in a 96-well plate at a density of 1 × 10^4 cells per well and supplemented with 25 or 100 IU/mL of IL-2 for 48 h. In the case of IL-2 (25 IU/mL) supplemented, NK-92 cells were treated with vitamin C (5, 10, and 20 μg/mL), aptamer (0.05, 0.1, and 0.2 μg/mL), or Aptamin C (5, 10, and 20 μg/mL). (A) Cell number and (B) viability were measured using the trypan blue dye exclusion assay. Data represent three independent experiments with values presented as means ± SD. * p<0.05, ** p<0.01, *** p<0.001.
